# HTA and HIV: The Case of Dual NRTI Backbones in the Italian Setting

**DOI:** 10.3390/ijerph17239010

**Published:** 2020-12-03

**Authors:** Elisabetta Garagiola, Emanuela Foglia, Lucrezia Ferrario, Giovanni Cenderello, Antonio Di Biagio, Barbara Menzaghi, Giuliano Rizzardini, Davide Croce

**Affiliations:** 1 Centre on Health Economics, Social and Health Care Management, LIUC Business School, LIUC-Università Cattaneo, 21053 Castellanza, Italy; efoglia@liuc.it (E.F.); lferrario@liuc.it (L.F.); dcroce@liuc.it (D.C.); 2 Department of Infectious Diseases, Galliera Hospital, 16128 Genova, Italy; g.cenderello@asl1.liguria.it; 3 Department of Infectious Diseases Unit ASL1- Imperiese, 18038 Sanremo, Italy; 4 Unit of Infectious Diseases, IRCCS San Martino IST Hospital, 16132 Genova, Italy; antonio.dibiagio@hsanmartino.it; 5 Department of Infectious Diseases, Valle Olona Hospital, 21052 Busto Arsizio, Italy; barbaramenzaghi@libero.it; 6 Department of Infectious Diseases, Fatebenefratelli Sacco Hospital, 20157 Milan, Italy; rizzardini.giuliano@asst-fbf-sacco.it; 7 Faculty of Health Sciences, School of Public Health, University of the Witwatersrand, Johannesburg 2193, South Africa

**Keywords:** HIV, dual NRTI backbones, HTA, Italy

## Abstract

The aim of this study is to analyze the potential advantages of emtricitabine/tenofovir alafenamide (FTC/TAF) introduction, creating evidence-based information to orient strategies to reduce costs, thus preserving effectiveness and appropriateness. An Health Technology Assessment (HTA) was implemented in the years 2017–2018 comparing the dual backbones available in the Italian market: FTC/TAF, FTC/TDF (tenofovir disoproxil fumarate/emtricitabine) and ABC/3TC (abacavir/lamivudine). From an efficacy point of view, FTC/TAF ensured a higher percentage of virologic control and a better safety impact than FTC/TDF (improving the renal and bone safety profile, as well as the lipid picture). From an economic point of view, the results revealed a 4% cost saving for the Italian National Healthcare Service NHS with FTC/TAF introduction compared with the baseline scenario. Qualitative perceptions’ results showed that FTC/TAF would decrease the burden of adverse events management, increasing the accessibility of patients to healthcare providers (FTC/TAF: 0.95, FTC/TDF: 0.10, ABC/3TC: 0.28; *p*-value: 0.016) and social costs (FTC/TDF: −0.23, FTC/TAF: 1.04, ABC/3TC: 0.23; *p*-value < 0.001), improving patient quality of life (FTC/TDF: 0.31, FTC/TAF: 1.85, ABC/3TC: 0.38; *p*-value < 0.001). Healthcare services may consider the evidence provided by the present study as an opportunity to include HIV patients in a more adequate antiretroviral treatment arm, guaranteeing a personalized clinical pathway, thus becoming more efficient and effective over time.

## 1. Introduction

The discovery of Highly Active Antiretroviral Therapies (HAART) and their introduction into clinical practice has transformed the human immunodeficiency virus (HIV) infection from a fatal condition to a chronic disease, decreasing morbidity and mortality as well as increasing life expectancy [1]. HAART has enabled the effective control of viral replication and immune status improvement in patients with HIV or acquired immunodeficiency syndrome (AIDS), leading to remarkable success in treatment [2]. Triple therapy with a 2-NRTI (Nucleotide Reverse Transcriptase Inhibitors) backbone plus a third agent (NRTI, Integrase Inhibitor—INI, or Protease Inhibitors—PI) is now considered the standard of care approach for HIV treatment [3].

In the HIV setting, innovation is currently playing a key role since treatment choices and management practices should ensure patients’ long-term health with minimal comorbidity [4]. This aspect is particularly important not only with regard to the introduction of once-daily fixed-dose co-formulations for reducing pill burden and maximizing long-term drug adherence, but also with regard to the advent of other 2-NRTI backbones [5]. These could present an increase in patients’ safety (with a consequent maximization of long-term tolerability in the context of earlier diagnosis, earlier initiation, longer duration of treatment and older age).

In this regard, the introduction of emtricitabine/tenofovir alafenamide (FTC/TAF) and the change of price setting of tenofovir disoproxil fumarate/emtricitabine (FTC/TDF) in Italy in 2017 has altered the HIV drug market which had been represented by triple therapy with the administration of FTC/TDF and abacavir/lamivudine (ABC/3TC). The rationale behind the development of TAF was to produce a therapeutic moiety with the potency of TDF combined with an improved safety profile [6]. While TDF therapy is generally well tolerated, it has been associated with effects on renal function and bone mineral density [7].

As a consequence, this has had an impact on policymaking processes, disinvestment strategies and allocation of economic resources. An information gap has emerged concerning the management of HIV patients. While HIV is a significant economic burden for healthcare providers, limited country-oriented evidence is available with regard to the resource absorption [8] or multidimensional evaluation. Therefore, an in-depth study is needed, particularly in the Italian national setting, where the government has launched a program of spending review aimed at cutting budgets and making the diffusion of technology-based healthcare innovations more difficult [9]. Regional governments evaluate and consider only the innovations that have been able to practically demonstrate their value for money.

Moving on from these premises, the aim of the proposed study is to analyze the potential advantages of FTC/TAF introduction creating evidence-based information to orient strategies useful to reduce costs in a context of budget cuts, also preserving effectiveness and appropriateness and guaranteeing universality and equity of care for HIV treatments, using a multi-dimensional approach (typically implemented in Health Technology Assessment (HTA) evaluation).

## 2. Materials and Methods

To achieve the abovementioned study objective, a complete HTA was implemented. The HTA approach aims at providing decision makers with relevant and reliable information in selecting technologies with the best value to cost ratio, considering evidence-based and multi-disciplinary methodologies to evaluate processes and a number of criteria that deal with their economic, social, clinical, ethical and organizational implications [10,11,12,13].

The introduction of healthcare technologies requires a deep analysis of direct expenditures and more attention to the related assessment, selecting only the most promising healthcare technologies, avoiding waste on innovations. HTA could support decision-making processes both at institutional–macro level and hospital–meso level. In the proposed analysis, the institutional National Healthcare Service perspective was taken into account.

The HTA strategy is a useful method to support the decision-making process, particularly considering investment or disinvestment. At the same time, HTA could support lean thinking and lean management, suggesting the area of optimization and improvement [14].

This HTA study was conducted in the years 2017–2018, comparing the dual NRTI backbones available in Italy: FTC/TAF, FTC/TDF and ABC/3TC, administered to both naïve and experienced HIV populations.

Due to the multi-dimensional nature of HTA, several aspects of these drugs were analyzed (using a comparative approach), as described in the EUnetHTA Core Model [15]: (i) general relevance of the healthcare problem; (ii) technical description of the technologies and potential benefits; (iii) safety; (iv) efficacy; (v) economic and financial impact; (vi) equity; (vii) legal aspects; (viii) social and ethical impact; and (ix) organizational implications 

The above dimensions were deployed, considering a literature review, economic and budget impact analyses [16] and qualitative approaches.

### 2.1. Literature Review

Before starting the assessment of the dimensions, the Problem/Population, Intervention, Comparator and Outcomes (PICO) approach [17] for the literature validation was identified (P—population: naïve and experienced HIV adult patients; I—intervention: TAF/FTC; C—comparators: TDF/FTC and 3TC/ABC; O—outcomes: Virologic control and adverse events).

The evidence in the literature came from a systematic search of databases (PubMed, Embase and Cochrane Library) from January 2011, to June 2018. The search terms included the following: “HIV treatment”, “emtricitabine/tenofovir alafenamide”, “disoproxil fumarate/emtricitabine”, “abacavir/lamivudine”, “clinical effectiveness”, “virologic control”, “drug-related adverse event”, “HIV naïve patients”, and “HIV experienced patients”. In accordance with the use of Grades of Recommendation, Assessment, Development, and Evaluation (GRADE) [18], within an HTA exercise on drugs, only randomized control trials (RCTs) were taken into consideration.

Peer-reviewed papers that explicitly described the clinical effectiveness and the safety profiles of TAF/FTC, TDF/FTC and 3TC/ABC were consequently included, and their quality was verified. The validation of the scientific evidence available on the topic was carried out by five experts (three HIV clinicians and two academic researchers, with an economic background. The professionals involved presented over ten years’ experience in the specific healthcare sector), using the approach suggested by both the Critical Appraisal Skills Programme (CASP) checklist [19] for RCTs, and IMPAQHTA Model [20].

For each selected study, the experts were asked to attribute first a qualitative synthesis of the results, and then, a score ranging from 4 (excellent) to 1 (insufficient) for the following items: (i) overall quality of the publication; (ii) generalizability of the results; (iii) completeness of the results.

The evidence in the literature, in terms of the occurrence rate of drug-related adverse events and of data regarding virologic control, was used for the presentation of both the safety and the efficacy dimensions related to EUnetHTA Core Model approach.

### 2.2. Economic and Budget Impact Analysis

To adequately assess the economic dimension, it was necessary, firstly, to analyze and determine the HIV clinical pathway cost as the average process costs absorption; and secondly, to verify the affordability of the economic results, developing a budget impact analysis [21].

The clinical pathway was assessed [22] considering the three different treatment strategies evaluated (FTC/TDF, 3TC/ABC or FTC/TAF), and was valorized according to the standard clinical procedure carried out by four Italian Infectious Disease Departments. The economic impact of a patient receiving FTC-/TDF-, 3TC-/ABC- or FTC-/TAF-based treatments, was determined using the components described below.

- Drugs’ costs (both HAART and other drugs): these were derived from the Italian ex-factory price updated to April 2018 and officially published on the NHS price list, considering the mandatory discounts required by law, and the VAT. For 3TC/ABC and TDF/FTC, the price of the generic drug published in the Italian Gazzetta Ufficiale (GU, the Ministerial Official Document, publishing all the approved drugs and related commercial prices [23]) was considered. For the economic evaluation of the complete therapeutic strategy, data derived from the Lombardy Region Clinical Pathway (CP) valid for the 2017 [24] were taken into account.
- Non-drug costs: other medical costs for HIV+ patients management, including the total amount of hematologic, cultural and laboratory tests, diagnostic procedures, outpatient and specialistic visits, medical examinations, and hospital admissions. The clinical pathway related to the abovementioned procedures administered to patients was defined according to Delphi methods [25], involving four infectious Disease Clinicians.
- Cost of side effect management: the clinical pathway on an annual basis for patients who develop specific adverse events was evaluated (in terms of laboratory tests, diagnostic tests, additional treatment and potential hospitalizations), as already described, by means of Delphi approach [25], thanks to the support of five infectious disease clinicians. After the definition of the percentage of adverse events occurring in the reference population (identified through evidence from the analysis of the available literature on the topic) and the standard clinical pathway resources absorption, the two variables were multiplied in order to define the overall economic impact of side effect management.

The economic analysis was performed with a time horizon of 12 months, assuming the National Healthcare Service perspective (third payer), according to the 2018 Italian Reimbursement Tariffs, for outpatients and hospital admissions, with a consistent approach with economic literature on the topic [26].

The economic evaluation of the HIV patient clinical pathway was analyzed on the basis of both treatment-naïve and treatment-experienced populations, hence also differentiated considering the viral control achieved or not by the patients (stratifying for viral load (VL) of ≤50 or >50 copies/mL at 48 weeks), as already reported in the economic literature on the field [27].

The process analysis of the HIV+ patients’ clinical pathways (stratified for populations characteristics) was conducted, and used also to perform a Budget Impact Analysis (BIA) using the above cost data inputs and showed the HIV healthcare evolution, up to three years. The BIA compared a baseline scenario, in which all eligible patients were treated with FTC-/TDF- and 3TC-/ABC-based regimens, with two different innovative scenarios (Table 1).

The first innovative scenario considered the introduction of FTC-/TAF-based regimens with the consequent complete disinvestment of FTC-/TDF-based regimens (except for patients who were pregnant or had epilepsy or tuberculosis, as suggested by clinical guidelines on the topic [28]). The second innovative scenario considered the introduction of FTC-/TAF-based regimen and FTC/TDF as a generic drug.

Market share considered for the development of the different scenarios derived from the Lombardy Region Clinical Pathway (CP), valid for the 2018, verified using the expert opinion of the Infectious Disease Department (Table 1).

After defining the comparative scenarios, the number of eligible patients was determined based on the Italian epidemiological data (prevalence and incidence rates), and epidemiological estimates regarding HIV [29]. In the first year, the BIA considered 86,454 HIV+ patients assuming a 2-NRTI backbone (equal to the 97% of the population presenting an HIV diagnosis). The incidence of the disease was 0.57% per year, and indicated the percentage of new patients entering in the budget impact model in the second and third year.

### 2.3. Qualitative Approach

Qualitative questionnaires derived from the issues proposed by EUnetHTA Core Model [15] were circulated among 21 healthcare professionals (18 clinicians and 3 pharmacists) referring to 18 Infectious Disease Departments across Italy. Six patients all with active participation in HIV+ Patients Associations were involved in the collection of individual perceptions, supported by qualitative questionnaire approach. This was useful to understand the current diffusion of the various alternatives investigated and the usage trends expected for the future.

Validated questionnaires were used to examine the qualitative EUnetHTA Core Model dimensions. The perceived impacts of the three therapeutic strategies were assessed in terms of (i) equity and accessibility aspects, (ii) social determinants, considering the patients’ perspective, (iii) legal impact and (iv) organizational factors, considering a comparative approach. The questions were phrased as statements: the healthcare professionals answered considering the level of agreement and accordance, with a 7-item Likert scale ranging from −3 to +3 (where −3 = absolutely negative/no accordance and +3 = absolutely positive/complete agreement), considering all the possible multi-dimensional impacts of the alternative drugs on the patients’ pathway. Data were analyzed considering descriptive statistics. One-way ANOVA tests were used to describe the existence of statistically significant differences between the three studied backbones. All analyses were conducted with a significant level of 0.05. All the analyses were performed using the statistical software SPSS 22.0 and MS Excel.

## 3. Results

### 3.1. Results from Literature Review

Out of the 1092 papers identified through database searching, only nine [30,31,32,33,34,35,36,37,38] met the inclusion criteria, in accordance with the search strategy focusing on the administration of FCT-/TDF-, FTC-/TAF- and 3TC/ABC-based regimens within naïve and experienced populations. The literature review revealed a lack of scientific evidence concerning head-to-head comparison between the three therapeutic regimens in terms of safety and clinical efficacy data.

Results deriving from the validation of literature were reported in Table 2: Although the number of studies presenting the comparison between FTC/TAF and FTC/TDF is wider, results suggested an equal balance in terms of literature validity for the three regimens (mean values: FTC/TAF 3.4 vs. FTC/TDF 3.3 vs. ABC/3TC 3.7).

The literature was used for efficacy and safety data retrieval (Table 3 and Table 4). From an efficacy point of view, FTC/TAF suggested an incremental percentage of virologic control if compared with FTC/TDF and ABC/3TC, both in case of treatment-naïve and treatment-experienced population.

Table 4 shows the drug-related adverse event incidence rates. Both FTC-/TAF- and FTC-/TDF-based regimens present a better safety profile than the ABC-/3TC-based regimen, especially in the treatment-naïve population. 

### 3.2. Results from the Economic and Budget Impact Analysis

The total cost of HIV+ patients management in one year, assuming the dual NRTI backbones, was evaluated (Table 5) considering the different patients’ characteristics.

In case of the change of price setting for FTC/TDF (whose cost was equal to EUR 1601.99), the total cost for a naïve patient was EUR 7690.37 in case of VL ≤ 50 copies/mL and EUR 8313.53 in case of VL > 50 copies/mL, whereas the economic evaluation for a treatment-experienced patient was equal to EUR 7867.50 in case of VL ≤ 50 copies/mL and EUR 8519.84 in case of VL > 50 copies/mL.

Table 6 reports the population entering the budget impact model and Table 7 synthesizes the final results deriving from the BIA. Considering 86,454 HIV+ patients, assuming the backbone and according to the economic evaluation presented in Table 5, the data revealed a 4.02% cost saving for the Italian NHS with FTC/TAF introduction compared with the baseline scenario, which could reach an 8.64% cost saving, with the contextual administration of the FTC/TDF (changing its price setting).

However, the introduction of FTC/TAF would require some additional investment in training courses (five days, on average, equal to EUR 3300) and meetings (3.5 h, on average), for a medium-sized hospital, with an Infectious Diseases Department.

### 3.3. Results from the Qualitative Approach

Focusing on the qualitative aspects (Table 8), based on the perceptions of the experts involved in the study, the results showed that FTC/TAF would decrease the burden of adverse events management: this fact led to an increase in the accessibility of patients to healthcare providers (FTC/TAF: 0.95, FTC/TDF: 0.10, ABC/3TC: 0.28; *p*-value: 0.016), with significant improvement in equity profile. Moreover, this fact implied also a freeing up of human resources (approximately at least 80 min per patients per year) due to the reduction of healthcare services for the monitoring of renal and bone profiles (on average, two blood samples and three diagnostic procedures); this time could be invested in the care of other HIV+ patients.

From a social perspective, FTC/TDF has a perceived negative impact on social costs (FTC/TDF: −0.23, FTC/TAF: 1.04, ABC/3TC: 0.23; *p*-value < 0.001), and patient quality of life (FTC/TDF: 0.31, FTC/TAF: 1.85, ABC/3TC: 0.38; *p*-value < 0.001), caused by long-term toxicities, considering patients’ perceptions of treatment outcome satisfaction.

With regard to the legal aspects, it should be noted that all the dual NRTI backbones in the study are mentioned in the Italian HIV National Guidelines [28]. Experts involved in the study considered FTC/TAF as a valuable therapeutic approach also in terms of legal impact (FTC/TAF: 1.13, FTC/TDF: 0.65, ABC/3TC: 0.73; *p*-value = 0.096), due to medicolegal issues in the prescription phase.

From an organizational perspective, FTC/TAF could simplify the overall management of HIV+ populations’ drug complications for hospitals (0.63 versus −0.14 (FTC/TDF) and 0.006 (ABC/3TC), *p*-value < 0.001).

## 4. Discussion and Conclusions

The HTA analysis provides clinicians, policymakers, and practitioners with evidence-based information useful to support HAART clinical choices in a general context of dynamic changing options for HIV treatment. Since HIV is a lifelong condition, its treatment needs to increasingly focus on the long-term management of patient complications and limiting treatment adverse effects, thus, also proposing simple dosages to reach a virologic control condition.

Clinical trials [33,34,35,36] reported that FTC-/TAF-based regimens would meet the above requirements, presenting a better safety profile than FTC-/TDF-based regimes.

Despite regimens based on FTC/TAF or FTC/TDF seeming approximately equivalent in effectiveness (in terms of achievement of adequate virologic control), it should be noted here that FTC/TAF could broaden the number of eligible HIV patients, particularly those at high risk of developing renal and bone disease, as well as adolescents who have not yet reached peak bone mass.

From an economic point of view, the results of the proposed study reported that the introduction of FTC/TAF could provide savings for the Italian NHS assuming a three-year time horizon. The lower medical costs associated with FTC-/TAF-based regimens reflect the reduction in adverse events management costs, with a higher cost decrease in treatment-experienced patients, compared with treatment-naïve patients, due to a higher development of serious adverse events.

Economic advantage related to adopting FTC-/TAF-based regimens would range from a minimum of 4% to a maximum of 8%, depending on the possibility to administer the FTC/TDF as a generic drug (even if this option would present significant drawbacks due to long-term onset of drug-related long-term toxicities).

The results of the study are consistent with scientific evidence available on the economic topic. An economic evaluation showed the economic superiority of FTC-/TAF-based regimens compared with FTC-/TDF- and ABC-/3TC-based regimens [7]. Additionally, two cost-effectiveness models developed in the Italian [39] and UK settings [37] reported that FTC-/TAF-based regimens would dominate the alternatives, being associated with better clinical outcomes and concomitant resource savings. 

Focusing on the other HTA dimensions, FTC/TAF would be the preferable therapeutic strategy depending on the patient’s perception of improved quality of life, and, in general, considering the perceived social impact (*p*-value = 0.000). Furthermore, the introduction of FTC/TAF would also generate organizational savings due to the reduction in the adverse events control activities, with the consequent freeing up of resources in terms of visits, laboratory tests, and overall management of complications. This positive organizational result could also consolidate the accessibility and equity profile of HIV+ treatments guaranteeing better care for the patients. This suggestion is also supported by the clinicians’ and patients’ perceptions, which are positive and incremental for FTC/TAF regimens (*p*-value = 0.016).

The introduction of FTC/TAF was found to deliver improvements in efficacy, with a greater chance of virologic control and safety compared with the other dual NRTI backbones, FTC/TDF and ABC/3TC. An FTC/TDF disinvestment strategy would be a cost-saving option for the Italian NHS.

To the best of authors’ knowledge, this study is the first attempt to fully evaluate the potential advantages of FTC/TAF introduction in clinical practice, with a multidimensional approach such as HTA methodology, offering new insights to advance the ongoing debate regarding the dual NRTI backbones given the paucity of economic resources.

The analysis, albeit conducted using real-world data and perceptions of the specific Italian setting, may have important implications for policymakers, since cost reduction is always priority, particularly in the current era of economic recession. Strategies are needed to reduce costs, preserving effectiveness and appropriateness, and guaranteeing universality and equity of care. Healthcare services may consider the evidence provided by the present study as an opportunity to include HIV+ subjects in the most adequate HAART treatment arm, guaranteeing a personalized clinical pathway, thus becoming more efficient and effective over time.

## Figures and Tables

**Table 1:** Market share of the three considered regimens in three different scenarios. Source: Expert opinion based on real-world evidence on consumption.

**Baseline Scenario (no Emtricitabine/Tenofovir Alafenamide FTC/TAF)**						
	**First year**		**Second year**		**Third year**	
	*Naïve*	*Experienced*	*Naïve*	*Experienced*	*Naïve*	*Experienced*
**TAF/FTC**	0.00%	0.00%	0.00%	0.00%	0.00%	0.00%
**TDF/FTC**	65.00%	65.00%	65.00%	65.00%	65.00%	65.00%
**3TC/ABC**	35.00%	35.00%	35.00%	35.00%	35.00%	35.00%
**First Innovative Scenario (with FTC/TAF)**						
	**First year**		**Second year**		**Third year**	
	*Naïve*	*Experienced*	*Naïve*	*Experienced*	*Naïve*	*Experienced*
**TAF/FTC**	52.50%	52.50%	66.00%	66.00%	77.00%	77.00%
**TDF/FTC**	12.50%	12.50%	9.00%	9.00%	8.00%	8.00%
**3TC/ABC**	35.00%	35.00%	25.00%	25.00%	15.00%	15.00%
**Second Innovative Scenario (Introduction of FTC/TAF and Tenofovir Disoproxil Fumarate/Emtricitabine FTC/TDF as a Generic Drug)**						
	**First year**		**Second year**		**Third year**	
	*Naïve*	*Experienced*	*Naïve*	*Experienced*	*Naïve*	*Experienced*
**TAF/FTC**	44.50%	44.50%	57.00%	57.00%	67.00%	67.00%
**TDF/FTC**	20.50%	20.50%	18.00%	18.00%	18.00%	18.00%
**3TC/ABC**	35.00%	35.00%	25.00%	25.00%	15.00%	15.00%

**Table 2:** Validation of the selected literature. Source: data gathered by authors based on expert opinion.

	TAF/FTC Based				TDF/FTC Based				3TC/ABC Based			
	Overall Quality	Generalizability	Completeness	Mean	Overall Quality	Generalizability	Completeness	Mean	Overall Quality	Generalizability	Completeness	Mean
**Mills et al., 2016**	3.6	3.6	3.4	3.5	4.0	3.0	3.4	3.5	NA	NA	NA	NA
**Gallant et al., 2016**	4.0	4.0	4.0	4.0	4.0	3.0	4.0	3.7	NA	NA	NA	NA
**Greig et al., 2016**	2.6	2.6	2.4	2.5	2.8	2.5	2.5	2.6	NA	NA	NA	NA
**Antela et al., 2016**	2.8	2.4	2.4	2.5	3.0	2.0	2.3	2.4	NA	NA	NA	NA
**Whol et al., 2016**	4.0	4.0	4.0	4.0	4.0	3.4	4.0	3.8	NA	NA	NA	NA
**Sax et al., 2015**	4.0	4.0	3.4	3.8	4.0	3.6	3.4	3.7	NA	NA	NA	NA
**De Jesus et al., 2017**	3.6	3.2	3.2	3.3	3.6	3.0	3.2	3.3	NA	NA	NA	NA
**Okin et al., 2017**	3.8	3.2	3.2	3.4	3.8	3.0	3.2	3.3	NA	NA	NA	NA
**Cruciani et al., 2014**	NA	NA	NA	NA	3.5	3.3	3.3	3.3	4.0	3.2	3.8	3.7
**Mean**	3.6	3.4	3.3	3.4	3.6	3.0	3.2	3.3	4.0	3.2	3.8	3.7

**Table 3:** Percentage of virologic control of the three regimens at 48 weeks.

	Naïve Population	Reference	Experienced Population	Reference
**TAF/FTC based**	92%	Antela et al., 2016	97%	Mills et al., 2016
**TDF/FTC based**	90%	Sax et al., 2015	93%	Gallant et al., 2016
**3TC/ABC based**	88%	Walmsley et al., 2013	93%	Sax et al., 2017

**Table 4:** Drug-related adverse event incidence rates of the three regimens and related economic evaluation, for 12-month clinical pathway.

**Treatment-Naïve Population**	**TAF/FTC–Based**	**Reference**	**TDF/FTC Based**	**Reference**	**3TC/ABC Based**	**Reference**	**Economic Evaluation of the 12-Month Clinical Pathway**
**Upper respiratory tract infection**	3.6%	Antela et al., 2016	3.1%	Antela et al., 2016	9%	Walmsley et al., 2013	EUR 199.88
**Diarrhea**	3.3%		2.5%		17%		EUR 168.45
**Headache**	2.9%		2.1%		13%		EUR 20.07
**Nausea**	2.2%		2%		14%		EUR 153.54
**Treatment-experienced population**	***TAF/FTC based***		***TDF/FTC based***		***3TC/ABC based***		***Economic evaluation of the 12-month clinical pathway***
**Upper respiratory tract infection**	9%	Gallant et al., 2016	11%	Mills et al., 2016	7.1%	Sax et al., 2017	EUR 199.88
**Diarrhea**	9%		10%	Gallant et al., 2016	12%		EUR 168.45
**Nasopharyngitis**	8%		6%		9.2%	Gallant et al., 2017	EUR 7.05
**Headache**	7%	Mills et al., 2016	8%	Mills et al., 2016	12.3%	Sax et al., 2017	EUR 20.07
**Cough**	6%	Gallant et al., 2016	5%	Gallant et al., 2016	2.5%	Gallant et al., 2017	EUR 10.05
**Syphilis**	5%	Mills et al., 2016	5%	Mills et al., 2016	7.9%		EUR 117.75
**Insomnia**	5%		6%		4.3%	Sax et al., 2017	EUR 277.30
**Arthralgia**	6%	Gallant et al., 2016	3%	Gallant et al., 2016	2.8%		EUR 148,71
**Bronchitis**	6%	Mills et al., 2016	5%	Mills et al., 2016	5.1%	Gallant et al., 2017	EUR 447.97
**Depression**	4%		5%		Not applicable		EUR 1002.10
**Osteopenia**	6%		6%		Not applicable		EUR 666.26
**Backache**	6%	Gallant et al., 2016	5%	Gallant et al., 2016	6.2%	Sax et al., 2017	EUR 527.90
**Nausea**	5%	Mills et al., 2016	3%	Mills et al., 2016	22.9%	Gallant et al., 2017	EUR 153.54
**Sinusitis**	4%	Gallant et al., 2016	5%		Not applicable		EUR 16.50
**Fatigue**	5%		4%	Gallant et al., 2016	8.6%	Gallant et al., 2017	EUR 22.50
**Vomit**	7%	Sax et al., 2015	6%	Sax et al., 2015	5.4%		EUR 52.74
**Rash**	6%		5%		Not applicable		EUR 320.65
**Pyrexia**	5%		5%		6.5%	Sax et al., 2017	EUR 232.97
**Dizziness**	5%		4%		Not applicable		EUR 113.31

**Table 5:** The total cost of HIV+ patient management, stratified for patients’ characteristics. Source: data gathered by authors.

Regimen	Virologic Control	Clinical History	Highly Active Antiretroviral Therapies HAART	Clinical Pathway	Adverse Events	Total
**TAF-/FTC-based regimen**	**Patient with VL ≤50 copies/mL**	**Treatment naïve**	EUR 8320.37	EUR 591.05	EUR 16.71	EUR 8928.13
		**Treatment experienced**	EUR 8320.37	EUR 1994.19	EUR 252.72	EUR 10,567.28
	**Patient with VL >50 copies/mL**	**Treatment naïve**	EUR 8320.37	EUR 768.18	EUR 16.71	EUR 9105.26
		**Treatment experienced**	EUR 8320.37	EUR 2200.50	EUR 252.72	EUR 10,773.59
**TDF-/FTC-based regimen**	**Patient with VL ≤50 copies/mL**	**Treatment naïve**	EUR 8926.49 *	EUR 1623.43	EUR 13.90	EUR 10,563.82
		**Treatment experienced**	EUR 8926.49 *	EUR 2025.16	EUR 248.93	EUR 11,200.58
	**Patient with VL ≤50 copies/mL**	**Treatment naïve**	EUR 8926.49 *	EUR 1800.56	EUR 13.90	EUR 10,740.95
		**Treatment experienced**	EUR 8926.49 *	EUR 2231.47	EUR 248.93	EUR 11,406.89
**3TC-/ABC-based regimen**	**Patient with VL ≤50 copies/mL**	**Treatment naïve**	EUR 5887.65	EUR 2017.71	EUR 70.73	EUR 7976.09
		**Treatment experienced**	EUR 5887.65	EUR 2121.30	EUR 173.83	EUR 8182.78
	**Patient with VL >50 copies/mL**	**Treatment naïve**	EUR 5887.65	EUR 2207.93	EUR 70.73	EUR 8166.31
		**Treatment experienced**	EUR 5887.65	EUR 2117.64	EUR 173.83	EUR 8179.12

* In case of the change of price setting of TDF/FTC, the HAART cost was considered equal to EUR 5980.24.

**Table 6:** Population considered in the Budget impact analysis. Source: data gathered by authors.

**Baseline Scenario (no FTC/TAF)**		**TAF/FTC**	**TDF/FTC**	**3TC/ABC**	**TOT**
	First year	-	56,195	30,259	86,454
	Second year	-	56,515	30,431	86,946
	Third year	-	56,837	30,604	87,441
	**Total**		**169,547**	**91,294**	**260,841**
**First Innovative Scenario (with FTC/TAF)**		**TAF/FTC**	**TDF/FTC**	**3TC/ABC**	**TOT**
	First year	45,388	10,807	30,259	86,454
	Second year	57,384	7825	21,737	86,946
	Third year	67,330	6995	13,116	87,441
	**Total**	**170,102**	**25,627**	**65,112**	**260,841**
**Second Innovative Scenario (Introduction OF FTC/TAF and FTC/TDF Generic Drug)**		**TAF/FTC**	**TDF/FTC**	**3TC/ABC**	**TOT**
	First year	38,472	17,723	30,259	86,454
	Second year	49,559	15,650	21,737	86,946
	Third year	58,585	15,739	13,116	87,441
	**Total**	**146,616**	**49,112**	**65,112**	**260,841**

**Table 7:** Budget impact analysis. Source: data gathered by authors.

**Baseline Scenario (no FTC/TAF)**		**TAF/FTC**	**TDF/FTC**	**3TC/ABC**	**TOT**
	First year	Not applicable	EUR 615,584,762	EUR 245,310,865	EUR 860,895,626
	Second year	Not applicable	EUR 619,087,985	EUR 246,706,901	EUR 865,794,887
	Third year	Not applicable	EUR 622,612,570	EUR 248,111,450	EUR 870,724,020
	**Total**	**Not applicable**	**EUR 1,857,285,317**	**EUR 740,129,216**	**EUR 2,597,414,533**
**First Innovative Scenario (with FTC/TAF)**		**TAF/FTC**	**TDF/FTC**	**3TC/ABC**	**TOT**
	First year	EUR 449,455,540	EUR 118,381,685	EUR 245,310,865	EUR 813,148,090
	Second year	EUR 568,245,343	EUR 85,719,875	EUR 176,219,215	EUR 830,184,433
	Third year	EUR 666,727,217	EUR 76,629,239	EUR 106,333,479	EUR 849,689,935
	**Total**	**EUR 1,684,428,100**	**EUR 280,730,799**	**EUR 527,863,559**	**EUR 2,493,022,458**
**Second Innovative Scenario (Introduction OF FTC/TAF and FTC/TDF Generic Drug)**		**TAF/FTC**	**TDF/FTC**	**3TC/ABC**	**TOT**
	First year	EUR 380,967,077	EUR 141,929,368	EUR 245,310,865	EUR 768,207,310
	Second year	EUR 490,757,342	EUR 125,330,112	EUR 176,219,215	EUR 792,306,669
	Third year	EUR 580,139,266	EUR 126,043,640	EUR 106,333,479	EUR 812,516,385
	**Total**	**EUR 1,451,863,685**	**EUR 393,303,121**	**EUR 527,863,559**	**EUR 2,373,030,365**
**Baseline Scenario vs. First Innovative Scenario**		**Not applicable**	**EUR −1,576,554,518** (−84.88%) *	**EUR −212,265,658** (−28.68%) *	**EUR −104,392,076** (−4.02%) *
**Baseline Scenario vs. Second Innovative Scenario**		**Not applicable**	**EUR −1,463,982,196** (−78.82%) *	**EUR −212,265,658** **(−28.68%)**	**EUR −224,384,169** **(−8.64%)**
**First Innovative Scenario vs. Second Innovative Scenario**		**EUR −232,564,415** (−13.81%) *	**EUR 112,572,322** (+40.10%) *	**EUR 0** (0.00%) *	**EUR −119,992,093** (−4.81%) *

* Negative values favored the innovative scenarios, considering the freeing up of resources.

**Table 8:** Qualitative analysis. Source: data gathered by authors.

**Equity Aspects**	**TAF/FTC**	**TDF/FTC**	**3TC/ABC**	***p*-Value**
Access to care on local level	1.96	1.52	1.85	0.443
Access to care for an individual with a legally protected status	1.6	1.45	1.6	0.923
Impact of HAART on the hospital waiting list	0.62	0.42	0.58	0.851
Impact of HAART on the access to care related to the management of mild and moderate adverse events (nausea, dizziness, headache or diarrhea)	0.81	0.38	0.23	0.148
Impact of HAART on the access to care related to the management of kidney problems	1.46	−1.15	0.69	0.000
Impact of HAART on the access to care related to the management of bone problems	1.37	−1.37	0.56	0.000
Impact of HAART on the access to care related to the management of cardiac problems	0.48	0.81	−0.48	0.001
Impact of HAART on the access to care related to the management of liver problems	0.7	0.04	−0.19	0.005
Impact of HAART on the access to care related to the management of long-term acute myocardial infarction development	1	0.81	−1.3	0.000
Impact of HAART on the access to care related to the management of long-term bone disease development	1.19	−1.19	0.41	0.000
Generation of health migration phenomena	0.77	−0.15	0.12	0.207
Existence of limiting factors in the use of HAART	0.22	−0.22	−0.33	0.196
HAART inequity	0.19	−0.04	−0.04	0.296
**Average Value**	**0.95**	**0.10**	**0.28**	**0.016**
**Social and ethical aspects**	**TAF/FTC**	**TDF/FTC**	**3TC/ABC**	***p*-value**
Ability of HAART to protect the patient’s autonomy	1.6	0.48	0.68	0.008
Ability of HAART to protect human rights	0.85	0.69	0.69	0.882
Ability of HAART to protect human integrity	0.96	0.62	0.62	0.506
Ability of HAART to protect the patient’s dignity	0.92	0.73	0.73	0.807
Ability of HAART to protect the patient’s religion	0.23	0.23	0.23	1.000
The use of HAART guarantees the willingness to pay of the patients	0.35	0.04	0.04	0.499
Impact of HAART on social costs	1.04	−0.23	0.23	0.000
Patients and citizens can have a good level of understanding of HAART	0.65	0.54	0.42	0.701
Impact of HAART on the easiness to be prescribed	1.15	0.38	0.38	0.035
Impact of HAART on the safety and the tolerability profile	1.69	−0.5	−0.04	0.000
Impact of HAART on the patient’s perceived quality of life	1.85	0.31	0.38	0.000
Impact of HAART on the caregiver’s life and perception.	1.15	0.5	0.62	0.062
Impact of HAART on the trusting relationship with the clinician	1.5	0.62	0.77	0.023
Impact of HAART on the patient’s satisfaction	1.68	0.48	0.68	0.000
Impact of HAART on the development of long-term adverse events and toxicity	1.58	−1.12	−0.62	0.000
Ethical impact of HAART insertion in the drug handbook	0.85	−0.05	0.5	0.029
**Average Value**	**1.13**	**0.23**	**0.4**	**0.000**
**Legal aspects**	**TAF/FTC**	**TDF/FTC**	**3TC/ABC**	***p*-value**
Need for HAART inclusion in the national or European registry	1.05	0.45	0.5	0.352
Need for HAART inclusion in national guidelines	1.7	1.2	1.3	0.408
Need for HAART inclusion in national clinical pathway	1	0.75	0.75	0.823
Legal problems related to the administration of HAART with a low safety and tolerability profile	1.05	−0.05	0.2	0.026
Need to regulate the acquisition of HAART	0.85	0.6	0.65	0.770
The legislation covers the regulation of HAART for all categories of patients	1.1	0.95	1	0.926
**Average Value**	**1.13**	**0.65**	**0.73**	**0.096**
**Organizational aspects**	**TAF/FTC**	**TDF/FTC**	**3TC/ABC**	***p*-value**
Additional staff	0.44	−0.11	0.17	0.257
Training course for the clinicians	0.5	0.39	0.33	0.931
Training course for healthcare professionals involved in the HAART distribution (nurses)	0.00	0.06	0.06	0.958
Training course for healthcare professionals involved in the HAART distribution (pharmacists)	0.39	0.22	0.22	0.846
Training course for other healthcare professionals involved in HAART administration	0.28	0.06	0.06	0.602
Patient and caregiver training	0.44	−0.06	−0.11	0.041
Hospital meetings required	0.5	0.17	0.22	0.653
Additional room space	−0.06	−0.06	−0.06	1.000
Additional furniture	0.06	0.00	0.00	0.375
Additional equipment	0.22	−0.11	−0.11	0.078
Impact of HAART on the internal processes	0.67	0.06	0.22	0.159
Impact of HAART on the purchasing processes	0.39	0.22	0.22	0.851
Impact of HAART on the hospital processes	0.17	−0.11	0.00	0.293
Impact of HAART on the access for monitoring visits and blood exams	1.61	−0.67	−0.11	0.000
Impact of HAART on the access for adverse events	1.11	−0.56	−0.06	0.000
Impact of HAART on the organizational management of adverse events	1	−0.56	−0.17	0.000
Impact of HAART on the organizational management of toxicity	0.83	−0.11	−0.17	0.007
Impact of HAART on the patient’s clinical pathway, in terms of management of kidney problems	1.28	−0.94	0.11	0.000
Impact of HAART on the patient’s clinical pathway, in terms of management of bone problems	1.39	−1.11	0.22	0.000
Impact of HAART on the patient’s clinical pathway, in terms of management of cardiac problems	0.83	0.56	−0.94	0.000
**Average Value**	**0.634**	**−0.140**	**0.006**	**0.000**
